# *Streptomyces rochei* ACTA1551, an Indigenous Greek Isolate Studied as a Potential Biocontrol Agent against *Fusarium oxysporum* f.sp. *lycopersici*

**DOI:** 10.1155/2013/387230

**Published:** 2013-05-20

**Authors:** Grammatiki S. Kanini, Efstathios A. Katsifas, Alexandros L. Savvides, Amalia D. Karagouni

**Affiliations:** National and Kapodistrian University of Athens, Faculty of Biology, Department of Botany, Microbiology Group, Panepistimioupolis, Zografou, 15781 Athens, Greece

## Abstract

Many studies have shown that several Greek ecosystems inhabit very interesting bacteria with biotechnological properties. Therefore *Streptomyces* isolates from diverse Greek habitats were selected for their antifungal activity against the common phytopathogenic fungus *Fusarium oxysporum*. The isolate encoded ACTA1551, member of *Streptomyces* genus, could strongly suppress the fungal growth when examined in antagonistic bioassays *in vitro*. The isolate was found phylogenetically relative to *Streptomyces rochei* after analyzing its 16S rDNA sequence. The influence of different environmental conditions, such as medium composition, temperature, and pH on the expression of the antifungal activity was thoroughly examined. *Streptomyces rochei* ACTA1551 was able to protect tomato seeds from *F. oxysporum* infection *in vivo* while it was shown to promote the growth of tomato plants when the pathogen was absent. In an initial effort towards the elucidation of the biochemical and physiological nature of ACTA1551 antifungal activity, extracts from solid streptomycete cultures under antagonistic or/and not antagonistic conditions were concentrated and fractionated. The metabolites involved in the antagonistic action of the isolate showed to be more than one and produced independently of the presence of the pathogen. The above observations could support the application of *Streptomyces rochei* ACTA1551 as biocontrol agent against *F. oxysporum*.

## 1. Introduction

Fusarium wilt diseases, including symptoms like wilting, chlorosis, necrosis, premature leaf drop, browning of the vascular system, stunting, and damping-off, were caused by pathogenic forma speciales of *Fusarium oxysporum*. These symptoms are the results of fungal spores and mycelium that block the xylem, toxin production and host defense responses such as tyloses, gums, and gels [1]. Fusarium wilting leads to severe losses in tomato and a variety of other crop plants [2].

*Fusarium oxysporum *f.sp.* lycopersici*, a soilborne plant pathogen in the class Hyphomycetes, causes Fusarium wilt specifically in tomato. This disease was first described by Massee in England in 1985 [3]. It is of worldwide economic importance as at least 32 countries, including Australia, Belgium, Canada, France, Germany, Greece, Israel, Italy, Japan, Mexico, Spain, Sweden, The Netherlands, UK, and USA, had reported the disease [4, 5] and results in severe losses in the greenhouses, open field crops, and hydroponic cultures.

Like all formae speciales of *F. oxysporum*, this fungus is a soil inhabitant and extremely difficult to control [6, 7]. However, several control methods have been used so far. Chemical treatments using benomyl, carbendazim, prochloraz, fludioxonil, bromuconazole, or azoxystrobin [8] not only result in fungicide resistant pathogens [9] but also cannot be characterized from a wide range of action while they are involved directly in the atmospheric pollution, undesirable effects on nontarget organisms, and possible carcinogenicity [10–14]. Additionally, soil amendments have been used in order to reduce the severity of the pathogen in crops by increasing the soil pH using fertilizers containing nitrate nitrogen [15] or by crop rotation [16, 17], but they have rarely provided long-term control in any production area. Currently, resistant cultivars have been used for the control of the disease [18, 19], but research was hampered by the lack of knowledge of the fungus genetic variability.

Thus, the above difficulties in controlling Fusarium wilt diseases have stimulated renewed interest in biological control as a disease management alternative [6]. For this purpose, root colonizing plant beneficial microorganisms have been used including species of *Pseudomonas (Pseudomonas fluorescens, P. putida, P. aureofaciens)* [20–24], *Bacillus (Bacillus subtilis, B. Polymyxa, and B. cereus) *[25–27], non-pathogenic *Fusarium* [28, 29], and Actinobacteria [30–33]. These microbes possess many traits that make them well suited as biocontrol and growth-promoting agents. These include the ability to (i) grow rapidly *in vitro* and to be mass produced; (ii) rapidly utilize seed and root exudates; (iii) colonize and multiply in the rhizosphere and spermosphere environments and in the interior of the plant; (iv) produce a wide spectrum of bioactive metabolites (i.e., antibiotics, siderophores, volatiles, and growth-promoting substances); (v) compete aggressively with other microorganisms and (vi) adapt to environmental stresses. The major weakness of pseudomonads as biocontrol agents is their inability to produce resting spores, which complicates formulation of the bacteria for commercial use [20]. *Bacillus* strains even if they are well equipped genetically to produce a vast array of antibiotics, only a limited part of this antibiotic-devoted genetic background may be readily expressed in soil and thus, only a part of this arsenal may be actually produced under natural conditions [34]. The nonpathogenic *Fusarium* strains need to be applied in large concentrations as the basic mode of action of these biocontrol agents is the competition of nutrients that leads to fungistasis (inhibition of chlamydospores germination). 

The species of the genus *Streptomyces* belong to the phylum of Actinobacteria characterized by a wide range of modes of action like antibiotic production, lysis of fungal cell walls; competition and hyperparasitism have been proved to be effective biocontrol agents either alone or in combination with other biocontrol agents. Several *Streptomyces* species such as *S. lydicus, S. lividans, S. olivaceoviridis, S. scabies, S. plicatus, S. hydroscopicus, S. violaceusniger, S. humidus, S. avermitilis, S. aureofaciens,* and *S. roseoflavus *are well-known producers of important compounds that are active against a wide variety of fungal pathogens [35]. These include a wide range of antibiotics as well as a variety of enzymes (i.e., chitinases), which degrade the fungal cell wall [35–40]. Metabolites from streptomycetes have been used in agriculture as growth promoters [41–44] and selected strains of the genus also have been used as direct biocontrol agents for other plant diseases [30, 45–48].

In this study, a total of 528 *Streptomyces* isolates from the Athens University Microbiology Laboratory Culture Collection were examined for their antifungal activity against *Fusarium oxysporum*. The measurement of their antifungal strength *in vitro* resulted in the selection of *Streptomyces rochei* ACTA1551 as the isolate that expressed the higher antagonistic activity. *Streptomyces rochei* ACTA1551 was used for further studies evaluating the potential of its use as biocontrol agent. We employed determination of the range and the optimal conditions of its antagonistic behavior, examination of culture extracts for antifungal activity, and studies to control the *Fusarium oxysporum in vivo*, using the plant *Lycopersicon esculentum* (Family: Solanaceae, Common name: Tomato) as a model target aiming to complete the profile of *Streptomyces rochei* ACTA1551 as biocontrol agent.

## 2. Materials and Methods

### 2.1. Microbial Strains

*Streptomyces* strains isolated from the rhizosphere of indigenous plants (Table 1(A)) and nonrhizosphere samples of special Greek habitats (Table 1(B)) were examined for their antifungal activity against *Fusarium oxysporum*. The streptomycetes were maintained as spore suspensions in 30% (w/v) glycerol at −20°C [49]. 

*Fusarium oxysporum *DSM62059 (*Fusarium oxysporum *Schlechtendahl: Fries f.sp. *lycopersici*, a laboratory strain provided by the DSMZ culture collection) was used as fungal target. The pathogenic fungal strain was grown on Potato Dextrose Agar (PDA) or in Potato Dextrose Broth (PDB) at 25°C and was maintained on PDA at 4°C.

### 2.2. Biocontrol Assay *In Vitro*

 Streptomycete aliquots (30 *μ*L) from a spore suspension in 30% (w/v) glycerol were used as inoculum for all *in vitro* antagonism bioassays. For the same test we used two full-loops of *F. oxysporum* mycelium from a 5-day-old culture on PDA.

 The ability of the *Streptomyces* isolates to inhibit the fungal growth was determined using an agar plate antagonism bioassay [33]. *F. oxysporum* inoculums was placed on either side of a two-days-old colony of each streptomycete. After incubation at 28°C for 5 days the inhibition zone was formed and its presence characterized the *Streptomyces* isolate as positive. 

 For the quantification of the antifungal activity the average (triplicate plates per strain × three independent experimental sets) of the quotient of the area of the inhibition zone, which was formed around and over the area of the streptomycete colony itself, was calculated (Antifungal activity = *πR*_*z*_^2^/*πR*_str_^2^ (*R*_*z*_ = radius of inhibition zone and *R*_str_ = radius of streptomycete colony)) [33].

### 2.3. Taxonomy of Streptomycetes

The selected isolate was further characterized through the amplification of its 16S rRNA gene. The 16S rDNA fragment was amplified by PCR using two universal primers [50, 51]: pA (5′-AGA GTT TGA TCC TGG CTC AG-3′) and R1492 (5′-TAC GGY TAC CTT GTT ACG ACT T-3′). Amplification reactions were performed in volumes of 50 *μ*L containing 40 ng template DNA, 0.4 *μ*M of each primer, 1X buffer with Mg^2+^, 1 unit of Taq DNA Polymerase (Biotools England) and 0.2 mM dNTPs. Nucleases free water was used to bring the reaction volume to 50 *μ*L. After initial denaturation at 95°C for 2 min, samples were cycled for 30 PCR cycles using the following cycle profile: 95°C denaturation for 30 sec, primer annealing at 53°C for 30 sec, and primer extension at 72°C for 2 min, plus a final 2 min elongation step at 72°C. Amplified PCR products were separated by gel electrophoresis on 1.2% (w/v) agarose gel and then purified using Nucleospin Extract PCR kit (Macherey-Nagel, Germany). The 16S rDNA fragment (>1400 bp) was fully sequenced (Macrogen, Republic of Korea) and the results were used for strain identification. For the detection of closest relatives, all sequences were compared with the BLAST function (http://www.ncbi.nlm.nih.gov/BLAST/). Sequence data were compiled using the MEGA 5.10 software [52] and aligned with sequences obtained from the GenBank (http://www.ncbi.nlm.nih.gov/) databases using the ClustalW aligning utility. Phylogenetic analysis was performed using neighbour joining method implemented in MEGA 5.10. One thousand bootstrap analyses (distance) were conducted.

### 2.4. Influence of Culture Conditions on Antagonistic Activity

The agar plate antagonism bioassay was repeated for the selected *Streptomyces* isolate using five different solid media; PDA (Potato Dextrose Agar, DSMZ media no. 129), AGS (Arginine Glycerol Salts, [49]), NA (Nutriend Agar, Biokar Diagnostics, Beauvais, France), CzA (Czapek Agar, [53]) and SAA (Streptomyces Antibiotic Agar, [53]), and four different pH values (pH 6, pH 7, pH 8, and pH 9). All media—pH combination plates were incubated at 26°C, 28°C, and 30°C for 2 days prior to fungal inoculation and five days after that. 

### 2.5. Biocontrol Assay *In Vivo*

For *in vivo* antagonism tests, a suspension of streptomycetes spores in Ringer 1/4 salt solution (NaCl 2.15 g/L, KCl 0.15 g/L, CaCl_2_ 0.075 g/L, K_2_HPO_4_ 0.5 g/L according to Wellington et al., 1990 [54]) (10^9^ spores per mL) was prepared from a 5-day-old culture on Arginine Glycerol Salts Agar (AGS), as described by Herron and Wellington (1990) [49] and used for the tomato seed treatments. *F. oxysporum* was cultured in Nutrient Broth (NB, Biokar Diagnostics, Beauvais, France) for 5 days at 28°C and 180 rpm. The mycelium was aseptically collected on filter paper and washed with three culture volumes of deionized-sterile water. Three grams of wet mycelium (dry weight 15 to 18% w/w) were resuspended in 1000 mL deionized sterile water and were thoroughly mixed with 1 kg of sterile soil [55].

*Streptomyces* isolate encoded ACTA1551 was selected for *in vivo* experiments due to the strong suppression it caused to *F. oxysporum* growth *in vitro*. A sandy silt loam soil (ASTM classification) with a pH of 7.9, taken from an area under intense agricultural exploitation in the Marathon area (42 km NE from the centre of Athens), was used. Prior to its use, the soil was air-dried in the dark at 22°C for at least 3 months, passed through a 2 mm sieve, and autoclaved twice (121°C, 60 min) on two separate days.

 Tomato seeds were sterilised for 30 minutes in a 20% chlorine suspension and then dried under sterile conditions. A number of sterile seeds were immersed into a suspension of streptomycetes spores in Ringer 1/4 salt solution [54] (10^9^ spores per mL) for 30 minutes and then dried under sterile conditions. Untreated sterile tomato seeds and sterile tomato seeds treated with the selected streptomycetes were planted in pots containing sterile soil either amended with *F. oxysporum* (3 g of wet washed mycelium per kg of soil) or not [55]. For every treatment, 20 seeds were planted in each pot (three replicates for each pot were prepared). Each full experiment was conducted in four different occasions, over a time period of eight months. The pots were incubated at 28°C under fluorescent light, and moisture was controlled daily at the level of 40% (w/w) for 25 days. The number of seeds that survived and/or germinated was evaluated in order to estimate the ability of the examined streptomycete to control the fungi *in vivo*. In addition, the height and weight of the emerged plants were measured for the estimation of the *in vivo* antagonism strength. 

### 2.6. Extraction and Fractionation of Streptomycete Metabolites from Solid Cultures

In parallel, the selected *Streptomyces* isolate was grown on SAA (Streptomyces Antibiotic Agar, [53]) since it was selected as optimum medium for high antifungal activity expression. The cultures were incubated at 28°C for 7 days. The medium around the formed streptomycete colony in a radius of 2 cm was cut off and blended for three minutes. The slurry was centrifuged at 4.000 g for 60 min and the cell free supernatant was collected. For the determination of antifungal activity, 400 *μ*L from the concentrated culture supernatant were placed into wells on SAA plates (formed using a cork borer—diameter 1 cm, depth 1 cm) that were inoculated with the fungus.

 Additionally, the inhibition zones of cocultures of the selected streptomycete ACTA1551 and *F. oxysporum *on SAA plates were removed and blended for three minutes. The slurry was collected by centrifugation as described above. After filtration, 400 *μ*L were placed in a similar manner into wells on SAA agar plates inoculated with the fungus for the determination of the antifungal activity. 

 All extracts from the solid cultures were fractionated into a high molecular weight (protein) and a low molecular weight (nonprotein) component on a Pharmacia PD-10 gel filtration column using de-ionized water for elution, according to the manufacturer's recommendation. Each fraction was concentrated by lyophilisation and examined for antifungal activity.

All the experiments performed were in three independent sets using triplicate plates.

### 2.7. Antimicrobial Activity of the Bioactive Extractions against Common Microbial Indicators

 400 *μ*L from the agar extracts of the five streptomycetes were placed into wells (diameter 1 cm) of agar plates (triplicate plates, three individual experimental tests) inoculated with *Escherichia coli, Bacillus subtilis, Pseudomonas fluorescens, *and* Saccharomyces cerevisiae*. The inhibition zones were observed and measured. 

### 2.8. Data Analysis

Statistical analysis for the various data sets was conducted through paired/unpaired *t*-tests, using SifmaStat/Plot software program (ver. 12.0; Systat Software Inc., Chicago, IL, USA). In all runs, a significance level of <0.05 was used. 

## 3. Results and Discussion 

### 3.1. Biocontrol Assay *In Vitro*

A total of 528 streptomycetes were isolated using selective media, assigned to the genus *Streptomyces *on the basis of their phenotypic characteristics. Isolates with inhibitory characteristics were selected and screened by the agar plate assay. The total percentage of antagonistic isolates was 7% (39 isolates out of the 528 tested could suppress the growth of *F. oxysporum*) (Table 1). 

 The sampling area that gave the higher percentage of isolates that could suppress the growth of *F. oxysporum* was the rhizosphere of evergreen woody shrubs from an island of the Aegean Sea but the strongest antifungal activity was observed by streptomycetes derived from the rhizosphere of *Pinus brutia* (Crete). The streptomycetes that were isolated from the rhizosphere of evergreen woody shrubs from an island of the Ionian Sea lacked any antifungal activity, whilst very low numbers of potential biocontrol agents rose from the rhizosphere of *Ceratonia siliqua* or the soil derived from cultivated area (Marathon, Attica District). 

 Although the percentage of the antagonistic isolates screened was low, these results in comparison with previous observations [33, 56–58] support the hypothesis that the Greek *Streptomyces* isolates are multiactive and promising for their application in several biotechnological processes, including biocontrol of soil borne fungal pathogens. 

 Focusing on the ecophysiological characteristics of the sampling areas that gave the most active isolates, we can conclude the following correlation: the Aegean Sea climate provides environmental stress on the indigenous *Streptomyces* populations as long as periods of high temperatures and drought are reflected in a soil poor in nutrients. Thus, the mentioned soil microorganisms with antagonistic properties seemed to be dominant. Furthermore the participation of the anthropogenic impact on the Aegean Sea islands, could probably explain the high percentages of isolates collected from the rhizosphere soil of the plant *Pinus brutia* (indigenous plant of Crete) and of the evergreen shrubs that were antagonistic to *F. oxysporum in vitro*. On the contrary, the very different climatic conditions of the soil of the rhizosphere of evergreen shrubs in the Ionian Sea islands (areas of high humidity and low anthropogenic impact) resulted in isolates which were not able to suppress the growth of the pathogenic target. 

**Figure 1:**
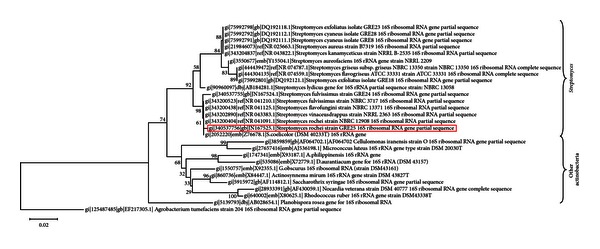
Phylogenetic tree of the 16S rDNA based on the neighbour-joining method, showing the position of the *Streptomyces rochei* ACTA1551. One thousand bootstrap analyses (distance) were conducted. The tree was rooted with *Agrobacterium tumefaciens*. Scale bar represents 2% estimated distance.

 The analysis of the above data resulted in the selection of the isolate encoded ACTA1551, derived from the rhizosphere soil of the plant *Pinus brutia* (indigenous plant of Crete) and expressed the highest antifungal activity (4.84 ± 0.05) of all the 39 isolates that were antagonistic against *F. oxysporum*. Analysis of 16S rRNA gene sequence data grouped the selected isolate to the species of *Streptomyces rochei *(99% identity with the 16S rDNA sequence of the closest phylogenetic relative, GenBank accession Number JN167525). The position of the selected streptomycete among the phylum of Actinobacteria is shown in Figure 1.

### 3.2. The effect of Nutrients, Temperature, and pH on Antagonistic Activity

**Figure 2:**
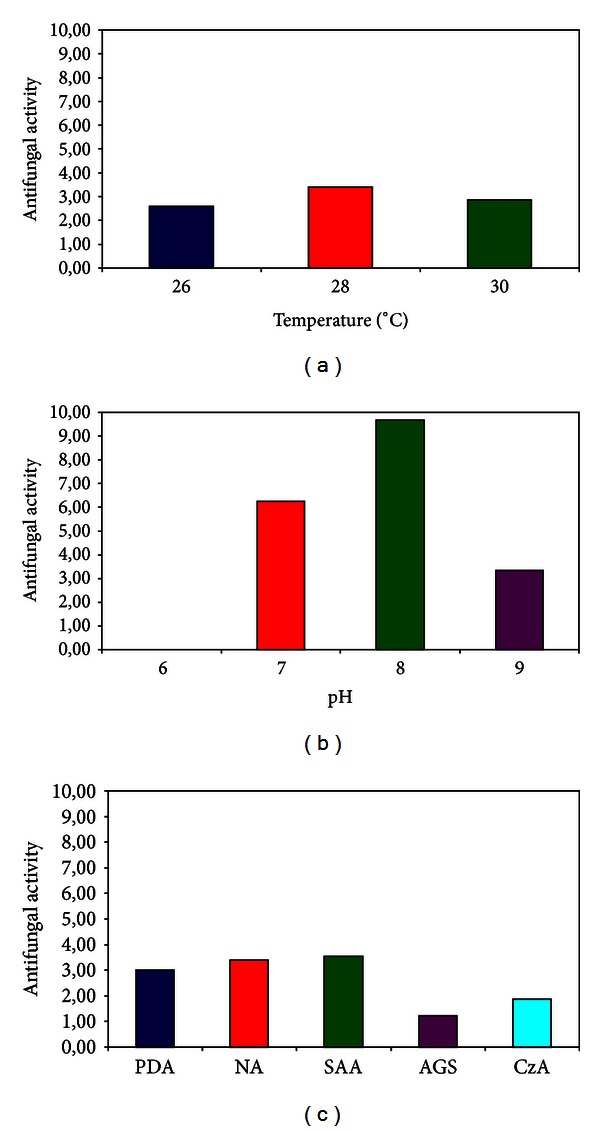
Influence of temperature, pH value, and media on the strength of antifungal activity expressed from ACTA1551 (*Streptomyces rochei*) on *Fusarium oxysporum*; (a) temperature effect, (b) pH effect, (c) carbon and nitrogen source effect.

Incubation of the selected streptomycete on SAA, containing glucose as carbon source and peptone from soya as nitrogen source, at 28°C and pH 8, was determined as the optimum culture conditions for maximum antagonistic activity against *F. oxysporum* (Figure 2). The temperature of 28°C is very close to the average of Mediterranean climatic temperature, while pH 8 is the value that characterizes the Greek cultivar soil. Therefore, one might suggest that the antifungal activity of the selected streptomycete ACTA1551 could be enhanced under the above conditions which are similar to real farming processes. 

### 3.3. Biocontrol Assay *In Vivo*

 Using the results from the *in vitro* antagonistic assay we were led to the selection of ACTA1551 (*Streptomyces rochei*) so as to use it for *in vivo* studies. This selection was based on its very high antifungal activity expressed *in vitro* (Table 2) and the observation from previous work [33, 56–60] that it is multiproducer of bioactive substances. Thus, it was characterized as promising biocontrol agent *in vivo*. 

 Analysis of particle size of the soil that was used for this purpose indicated the presence (%, dry weight) of sand, 50; silt, 36; clay, 14. Mineralogy analysis showed the presence of (%, dry weight) illite, 65; chlorite, 7; kaolinite, 10; smectite, 12; talc, 6 and calcite <1. Phosphorus content was 124 mg/L dry soil and organic carbon was 1.23% (dry weight). 

 The first evidence of the effective usage of ACTA1551 as biocontrol agent *in vivo *came from macroscopic observations. The *F. oxysporum* infected soil, where untreated tomato seeds were planted, was fully cover by the white mycelium of the fungi, while only limited white spots of fungal mycelium were observed onto infected soil where ACTA1551 treated tomato seeds were planted.

Considering the emergence of the tomato plants, the *in vivo* experiments showed that the tomato seeds treated with ACTA1551 germinated in statistically significant (*P* < 0.001) higher rates into *F. oxysporum* infected soil compared to untreated seeds. Even if the germination of ACTA1551 treated tomato seeds was lower than the positive control treatment (untreated tomato seeds planted into noninfected soil), in a statistically significant level (*P* < 0.001), it is clearly higher compared to the results when ACTA1551 was absent. These results provide evidence for an effective antifungal behavior of the selected streptomycete *in vivo*. However, the presence of ACTA1551 on the tomato seeds couldnot enhance the germination rate when planted in noninfected soil (Table 2).

**Figure 3:**
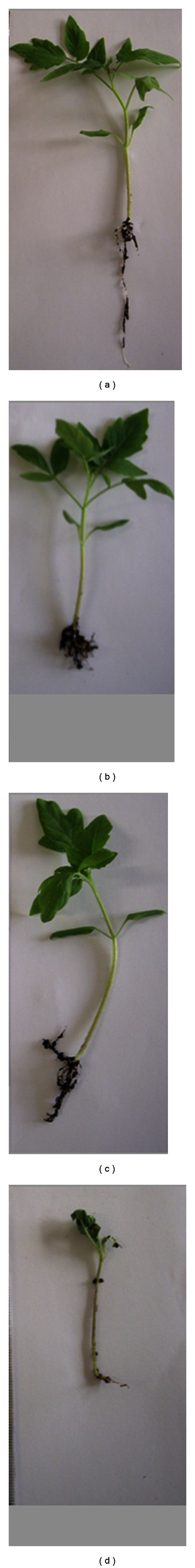
*In vivo* antifungal ability of ACTA1551 (*Streptomyces rochei*). Growth of the tomato plants (from left to right). (a) Untreated seed planted in untreated sterile soil (positive control), (b) seed treated with ACTA1551 (*Streptomyces rochei*) and planted in *F. oxysporum* infected soil, (c) seed treated with ACTA1551 (*Streptomyces rochei*) and planted in *F. oxysporum* noninfected soil, (d) untreated seed planted in *R. solani* DSM843 infected soil (negative control).

The analysis of the estimated values of mean weight and height of the emerged tomato plants showed that although the number of untreated plants that emerged in infected soil was low, the plants themselves were statistically significantly higher and stronger than the plants emerged from untreated seed in infected soil (*P* < 0.001). The treatment of the seeds with ACTA1551 enhanced the weight and the height of the plants compared to the negative control (untreated tomato seeds planted into infected soil). These results showed the ability of ACTA1551 to protect tomato seed from the pathogenic effects of *F. oxysporum *(Table 3, Figure 3).

Moreover, the *in vivo* results for noninfected soil showed that the presence of ACTA1551 produced plants of statistically significantly higher mean values of weight and height (*P* ≤ 0.001) compared to the positive controls (untreated tomato seeds planted into noninfected soil). The presence of ACTA1551 on the seeds promoted the growth of the emerged plants as the height of the treated plants in noninfected soil was greater than the height of untreated plants in noninfected soil and the treated plants were heavier than the untreated, in both cases, in a statistically significant level (*P* ≤ 0.001) (Table 3, Figure 3).

The above results provided strong evidence that ACTA1551 could protect tomato plants from wilt symptoms caused from *F. oxysporum* under the studied conditions and are in agreement with those from previews studies on the potential of *Streptomyces* isolates to be used as biocontrol agents. For instance, Reddi and Rao [61] reported that isolates of *Streptomyces ambofaciens* were able to control *Pythium* damping-off in tomato plants and *Fusarium* wilt in cotton plants, in an artificially infested soil while Mahadevan and Crawford [62] found that *Streptomyces lydicus* was identified as a broad spectrum biocontrol agent and the work of Farrag [63] enhanced that finding. 

Sterilization of soils by pasteurisation, fumigation, or autoclaving usually allows the pathogen to proliferate giving the opportunity to the biocontrol agent to express its antagonistic activity under optimal conditions for the target's growth. Thus, the results from these experiments clearly suggested the antagonistic relationship between the pathogen and the biocontrol agent. Other observations have noted that in nonsterile soils the native microflora slowed up the growth of the pathogen giving the opportunity to the biocontrol agent to express its antimicrobial activity more effectively [64]. Of additional importance, is that through our experimental approach, the streptomycete can protect the plant just by simple coating of the seeds with spore suspension prior to sowing. This inoculation method has been proved more effective as the biocontrol agent can rapidly and extensively cover the surface of the seeds [33, 55]. Early colonization by a biocontrol agent often is required to fill the critical niches and to effectively compete against pathogenic fungi [65]. Thus, seed coating with bacterial and fungal biocontrol agents often is utilized or required to control aggressive, rapidly growing soil-borne pathogens [65, 66]. Additionally, this procedure is much easier to implement and more applicable for large scale cultivation, compared to the classic one which includes enrichment of the soil with the biocontrol agents which is both time consuming and difficult to apply in real farming conditions [48, 67]. However, experiments of a larger scale under greenhouse conditions or in the field should support this potential in a more applicable way. The undeniable fact is that ACTA1551 strain meets the conditions of a promising biocontrol agent. 

Additionally, the same results give some information about the mechanism of action of the selected streptomycete in soil. As it was shown, ACTA1551 could promote the growth of the tomato plants in statistically significant levels, when it was the only microorganism in the soil, leading the tomato plants to overcome the growth levels they reached with no pathogen in their environment. Therefore, the possibility of acting as a growth promoter could raise further examination.

### 3.4. Extraction and Fractionation of Streptomycetes Metabolites from Solid Cultures

**Figure 4:**
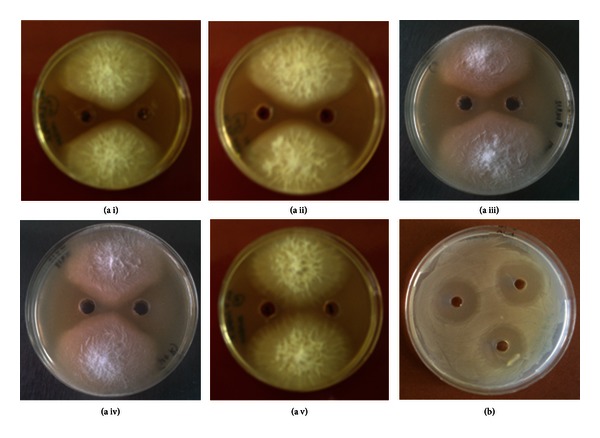
(a) Antifungal activity of the concentrated medium extracts derived from the solid culture of *Streptomyces rochei* ACTA1551 with or without *F. oxysporum*. Inhibition zone caused from (i) *Streptomyces rochei* ACTA1551 culture extract when *F. oxysporum* is present, (ii) *Streptomyces rochei* ACTA1551 culture extract when *F. oxysporum* is absent, (iii) *Streptomyces rochei* ACTA1551 low molecular weight fractions, (iv) *Streptomyces rochei* ACTA1551 high molecular weight fractions and negative control (no streptomycete extract added) (v). (b) Antifungal activity of the concentrated medium extracts derived from the solid culture of *Streptomyces rochei* ACTA1551 against *E. coli*.

 The concentrated extracts from the agar inhibition zones of the selected *Streptomyces* strain ACTA1551 (*Streptomyces rochei*) could strongly suppress the growth of *F. oxysporum* (Figure 4(a)). Additionally, the same antifungal activity was observed from the extracts of the solid culture of ACTA1551 without the presence of *F. oxysporum*. This observation may suggest that the streptomycete does not require the target fungus in its area to induce the production of the antifungal substances. The antifungal activity is fungus independent and the antifungal components may belong to the main metabolism of *Streptomyces rochei* ACTA1551.

 Following fractionation of the bioactive extracts in a gel filtration column, the antifungal activity was observed in both the low molecular weight and the high molecular weight fractions (Figure 4(a)). These results provide a combined action of different antifungal compounds. Each of them can independently suppress the growth of the pathogen *in vitro*, but their combination (crude culture extract) can enhance the antifungal action. Although, further biochemical analysis, using High Performance Liquid Chromatography fractionation coupled with Mass Spectrometry and Nuclear Magnetic Resonance techniques will allow us to refine its structure.

Moreover, examining the antimicrobial activity of the bioactive extractions against common microbial indicators (*Escherichia coli, Bacillus subtilis, Pseudomonas fluorescens, *and* Saccharomyces cerevisiae*) it was shown that the agar extract from *S. rochei* (ACTA1551) was able to inhibit growth of all the examined indicator microorganisms (Figure 4(b)). Therefore the antifungal activity of the selected streptomycete has a wider antimicrobial activity.

## 4. Conclusion

This study focused on screening the special and biotechnologically interesting Greek *Streptomyces* microflora in order to detect strains able to control the phytopathogenic fungi *Fusarium oxysporum*. The best antifungal producer, *Streptomyces rochei* ACTA1551 from the rhizosphere soil of the plant *Pinus brutia* (indigenous plant of Crete), proved to be capable of protecting tomato plants from Fusarium wilting under laboratory conditions while evidence that it promoted the plants growth was derived. The same streptomycete strain showed to be a promising producer of metabolites of a wide range of antimicrobial activity. Therefore, the present study demonstrated that *Streptomyces rochei* ACTA1551 is a new potential biocontrol agent at least against the pathogenic fungus *Fusarium oxysporum*.

## Figures and Tables

**Table 1:** *Streptomyces* strains with antifungal activity originated from twelve studied Greek habitats.

Sampling area	Number of isolates tested	Number (percentage) of isolates with antifungal activity against *F. oxysporum *DSM62059 {highest; lowest; average}^1^
(A) Rhizosphere samples		
(1) Rhizosphere of *Ebenus sibthorpii *	28	3 (10.7%) {2.36; 1.3; 1.5}
(2) Rhizosphere of *Ceratonia siliqua *	42	1 (2.4%) {1.96; 1.96; 1.96}
(3) Rhizosphere of *Olea europea *	73	6 (8.2%) {2.57; 2.13; 1.34}
(4) Rhizosphere of *Pinus brutia* from Crete	20	3 (15.0%) {4.84; 3.26; 1.56}
(5) Rhizosphere of evergreen woody shrubs from an island of the Aegean Sea	21	5 (23.8%) {2.69; 1.87; 1.47}
(6) Rhizosphere of evergreen woody shrubs from an island of the Ionian Sea	28	0 (0.0%)
(7) Rhizosphere of coniferous trees (Arcadian forest)	84	6 (7.1%) {2.91; 2.05; 1.56}
Rhizosphere subtotals	**296**	**21 (7.0%)**
(B) Nonrhizosphere samples		
(8) Hot spring water of Thermopiles thermal springs (Viotia district)	22	2 (9.1%) {1.63; 1.46; 1.28}
(9) Sediment from a volcanic area (Santorini island—Aegean sea)	5	1 (20%) {1.3; 1.3; 1.3}
(10) Soil derived from cultivated area (Marathon, Attica district)	179	10 (5.6%) {2.25; 1.6; 1.25}
(11) Soil from protected natural forest area (Kessariani, Attica District)	26	2 (7.7%) {1.39; 1.37; 1.34}
Nonrhizosphere subtotals	**232**	**15 (6.0%)**

Total	**528**	**39 (7.0%)**

^1^Antagonistic activity levels as expressed by the quotient of the inhibition zone area over streptomycete colony area (See Section 2.2).

**Table 2:** Germination data of tomato seeds during *in vivo* antagonism bioassays. The total number of planted tomato seeds was (20 per pot) × (three replicates for each pot) × (four independent experiments) = 240. All experimental data were combined for the analysis.

	Soil infected with* Fusarium oxysporum *			Soil not infected with* Fusarium oxysporum *		
	Number of planted tomato seeds	Number of germinated tomato seeds	Germination percentage^1^	Number of planted tomato seeds	Number of germinated tomato seeds	Germination percentage^1^
Seeds treated with ACTA1551	240	181 ± 2	75%	240	218 ± 2.2	90%
Untreated tomato seeds	240	108 ± 1	45%	240	228 ± 1.9	95%

^1^Unpaired *t-*test on the number of germinated seeds: seeds treated with ACTA1551 in infected soil versus untreated tomato seeds in infected soil were significantly different (*P* < 0.001); seeds treated with ACTA1551 in noninfected soil versus untreated tomato seeds in noninfected soil were significantly different (*P* < 0.001).

**Table 3:** Mean height and weight data of tomato plants derived from emerged tomato seeds during *in vivo* antagonism bioassays. The total number of planted tomato seeds was (24 per pot) × (three replicates for each pot) × (four independent experiments) = 288. All experimental data were combined for the analysis.

	Soil infected with *Fusarium oxysporum *		Soil not infected with *Fusarium oxysporum *	
	Mean height (cm)^1^	Mean weight (g)^2^	Mean height (cm)^1^	Mean weight (g)^2^
Seeds treated with ACTA1551	13.42 ± 0.1	0.58 ± 0.01	19 ± 0.2	0.79 ± 0.01
Untreated tomato seeds	11.16 ± 0.1	0.35 ± 0.01	15.01 ± 0.1	0.51 ± 0.01

^1^Unpaired *t-*test on *plant heights*: seeds treated with ACTA1551 in infected soil versus untreated tomato seeds in infected soil were significantly different (*P* < 0.001); seeds treated with ACTA1551 in noninfected soil versus untreated tomato seeds in noninfected soil were significantly different (*P* < 0.001).

^2^Unpaired *t-*test on *plant weights:* seeds treated with ACTA1551 in infected soil versus untreated tomato seeds in infected soil significantly different (*P* < 0.001); seeds treated with ACTA1551 in noninfected soil versus untreated tomato seeds in noninfected soil were significantly different (*P* < 0.001).
